# Three-dimensional characteristics of temporomandibular joint morphology and condylar movement in patients with mandibular asymmetry

**DOI:** 10.1186/s40510-022-00445-0

**Published:** 2022-12-29

**Authors:** Lin Tun Oo, Jun J. Miyamoto, Jun-Ichi Takada, Shih-Wei Eric Cheng, Hideyuki Yoshizawa, Keiji Moriyama

**Affiliations:** Department of Maxillofacial Orthognathics, Division of Maxillofacial and Neck Reconstruction, Graduate School of Medical and Dental Sciences, Tokyo Medical and Dental University (TMDU), 1-5-45 Yushima, Bunkyo-Ku, Tokyo, 113-8549 Japan

**Keywords:** Condylar movement, Mandibular asymmetry, Temporomandibular joint, Glenoid fossa, Condyle

## Abstract

**Background:**

Investigating the morphological and functional effects on mandibular asymmetry (MA) is important not only to understand the developmental process of masticatory dysfunction, but also to provide suggestions for evidence-based occlusal treatment.

**Aim:**

To evaluate three-dimensional temporomandibular joint (TMJ) morphology and its relationship to asymmetrical condylar movement in MA patients.

**Materials and methods:**

Fifty subjects were divided into MA and control groups (*n* = 25 each) according to a menton deviation of 4 mm from the mid-sagittal plane. TMJ morphology (condyle, glenoid fossa and TMJ spaces) were evaluated using a three-dimensional analysis programme. Three-dimensional condylar movements (from the sagittal and horizontal planes) were recorded and measured by computerized axiography on protrusion. Side-to-side asymmetry was measured for each parameter. The asymmetry index value was calculated to assess the correlation between TMJ morphology and condylar movement. For the statistical analysis, Wilcoxon’s signed-ranked test, the Mann–Whitney U test, and Spearman’s rank correlation were used.

**Results:**

Glenoid fossa volume, surface area, anteroposterior length, and condylar volume were significantly smaller, and articular eminence angle, glenoid fossa, and condylar axial angle were significantly larger, on the shifted side of the MA group when compared with those on the non-shifted side and the mean values of the control group (*P* < 0.05). The TMJ spaces of the MA group showed no bilateral difference but were significantly narrower in the medial, superior, and anterior joint spaces when compared with the control group (*P* < 0.05). Condylar path length and sagittal condylar inclination were significantly asymmetrical. The asymmetry index of the condyle volume was significantly correlated with that of the condylar path length (*P* = 0.005). The asymmetry index of the glenoid fossa volume and the articular eminence angle were significantly correlated with that of the sagittal condylar inclination (*P* = 0.009 and *P* = 0.002, respectively), and the asymmetry index of glenoid fossa volume was significantly correlated with the bilateral transverse condylar inclination (*P* = 0.006 and *P* = 0.016, respectively).

**Conclusions:**

Morphological asymmetry of the TMJ is significantly different between the shifted and non-shifted sides and is closely related to functional asymmetry of condylar movement in MA patients. (350/350).

**Supplementary Information:**

The online version contains supplementary material available at 10.1186/s40510-022-00445-0.

## Introduction

In patients with mandibular asymmetry (MA), craniofacial structures, including the glenoid fossa, condyle, and mandible, differ bilaterally in size and morphology [1]. Most previous studies have focused only on asymmetry of the condyle and mandible when considering craniofacial asymmetry [2–4]. However, the morphology of the glenoid fossa is reported to correlate with craniofacial morphology [5], and alteration in the articular eminence inclination has an effect on the growth changes in the morphology of the condyle and mandible [6].

In addition to bilateral differences in the craniofacial structures, functional asymmetry (i.e. asymmetrical masticatory dysfunction such as asymmetrical condylar movement and muscle activity) has also been reported in MA [7–9]. Studies conducted on rats showed that the lateral displacement of the mandible with asymmetrical masticatory dysfunction causes asymmetrical changes in the glenoid fossa and condyle, resulting in asymmetry of the mandible [10, 11]. Given that masticatory dysfunction can cause changes in the morphology of both the glenoid fossa and the condyle, and the morphology of the glenoid fossa is closely related to mandibular growth, elucidating the relationship between morphological and functional asymmetry is considered important in investigating the aetiology of MA. In addition, although these functions improved symmetrically after surgery, the asymmetry remained post-surgically [8, 12, 13]. The residual asymmetry of muscle activity is suggested to be associated with relapse [12, 13]. Meanwhile, asymmetrical condylar movement was also reported to remain after surgery and was speculated to be affected by the morphological asymmetry of the glenoid fossa [8]. However, it remains to be confirmed whether the postoperative functional asymmetry of condylar movement resulted from compensation for the anatomical asymmetry of the glenoid fossa, leading to postoperative relapse of mandibular asymmetry.

On the basis of the above, understanding the relationship between the asymmetry of the TMJ structure and the masticatory function of condylar movement is considered important, not only to comprehensively understand the developmental process of MA and masticatory dysfunction, but also to provide suggestions for evidence-based occlusal treatment in MA patients. To investigate the morphological and functional effects of MA, the current study evaluated the three-dimensional (3D) morphology of the TMJ structure including the glenoid fossa, condyle, and joint spaces and their relationship to asymmetrical condylar movement in MA patients.

## Materials and methods

Patients who received 3D computed tomography (CT) evaluation for orthognathic surgical planning by oral surgeons were included in this retrospective analysis. We excluded skeletal Class II patients because the TMJ morphology in these patients differed significantly from other skeletal class patients (i.e. skeletal Class I and III patients) [14, 15]. Then, we selected 50 adult patients with an ANB of < 2°. Patients with congenital malformations, such as cleft lip and palate, fractures, TMJ pain, or functional lateral mandibular shift, were excluded. The participants provided fully informed consent in accordance with the protocol approved by our university’s institutional ethics committee (approval number 731).

**Fig. 1:**
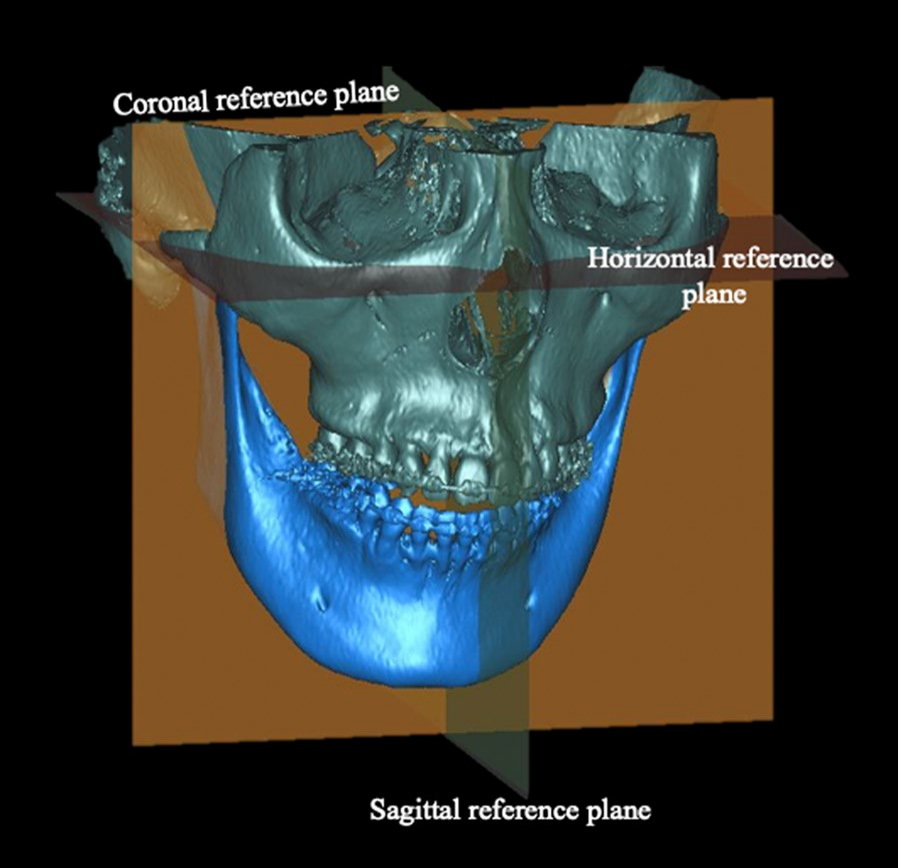
Three-dimensional computed tomography image of a patient with mandibular asymmetry with menton deviation greater than 4 mm from the mid-sagittal plane

On 3D images, MA was defined as a menton deviation of more than 4 mm from the mid-sagittal plane as characterized by Tun Oo et al. [16] (Fig. 1). Subjects with a menton deviation ≥ 4 mm were defined as the MA group (MA group; *n* = 25; 14 women, 11 men). Subjects with a menton deviation of < 4 mm were defined as the control group (*n* = 25; 14 women, 11 men). In the power analysis, the sample size was estimated to be at least 18 in each group with total of 36 patients, for a power calculation of 0.80 and an alpha of 0.05 (G*Power, version 3.1.9.6).

The CT scans were performed using the following parameters on a multi-slice CT unit (SOMATOM PLUS-S; Siemens Japan, Tokyo, Japan): 3-mm slice thickness, 4 mm per second table speed, 120 kV, and 200 mA. Sim-plant Pro (version 13; Materialise Dental NV, Leuven, Belgium) was used to recreate the CT images’ digital imaging and communication in medicine (DICOM) files into 3D images. Anatomical landmarks were directly marked on the 3D images in accordance with the method used in previous studies [16, 17], and the reference planes were constructed as reported by Tun Oo et al*.* [16] (Table 1).

**Table 1:** Definitions of anatomical landmarks and reference planes

Landmark	Definition
Or (Infraorbitale)	Most inferior point of the bony orbitale
Mid-Or	Middle point of the bilateral orbitale
Po (Porion)	Most superior point of the external auditory meatus
FoS	Centre of the foramen spinosum
Mid-FoS	Middle point of the bilateral foramen spinosa
Gf-Sup	Most superior point of the glenoid fossa
AT (Articular tubercle)	Most inferior point of the articular tubercle
ENP (Entoglenoid process)	Most inferior point of the entoglenoid process
POP (Postglenoid process)	Most inferior point of the postglenoid process
Gf-Ant	Most anterior point of the base of glenoid fossa perpendicular to ENP-AT line and passing POP
Cd-Lat	Most lateral point of the condyle
Cd-Med	Most medial point of the condyle
Cd-Ant	Most anterior point of the condyle
Cd-Post	Most posterior point of the condyle
Cd-Sup	Most superior point of the condyle
Me (Menton)	Most inferior midpoint of the symphysis
Reference planes	
Horizontal reference plane (FH plane)	Plane passing through the right and left porion and the middle point of the bilateral orbitale
Sagittal reference plane	Perpendicular to the FH plane and passing through the middle point of the bilateral foramen spinosa and the middle point of the bilateral orbitale
Coronal reference plane	Perpendicular to the FH plane and sagittal reference plane and passing through the midpoint of the bilateral foramen spinosa
Fossa base plane	Plane passing through the AT, ENP and POP

**Fig. 2:**
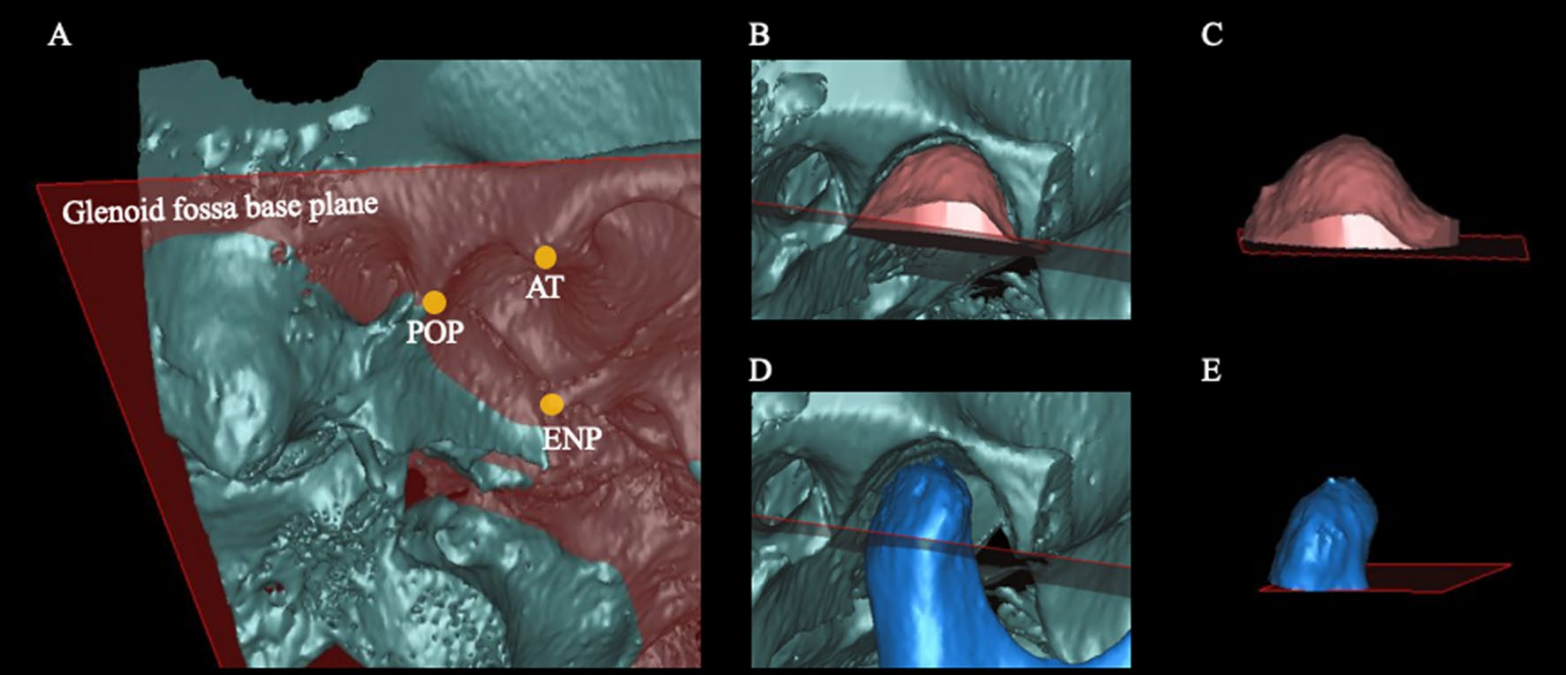
Evaluation of the glenoid fossa and condyle volume. **A**: The glenoid fossa base was defined as the plane passing through the most inferior point of the articular tubercle (AT), the most inferior point of the entoglenoid process (ENP), and the most inferior point of the postglenoid process (POP). **B** and **C**: The glenoid fossa volume and glenoid fossa surface area were measured for the structure above the glenoid fossa base. **D** and **E**: The condyle volume was defined as the volume of the condyle above the glenoid fossa base

**Fig. 3:**
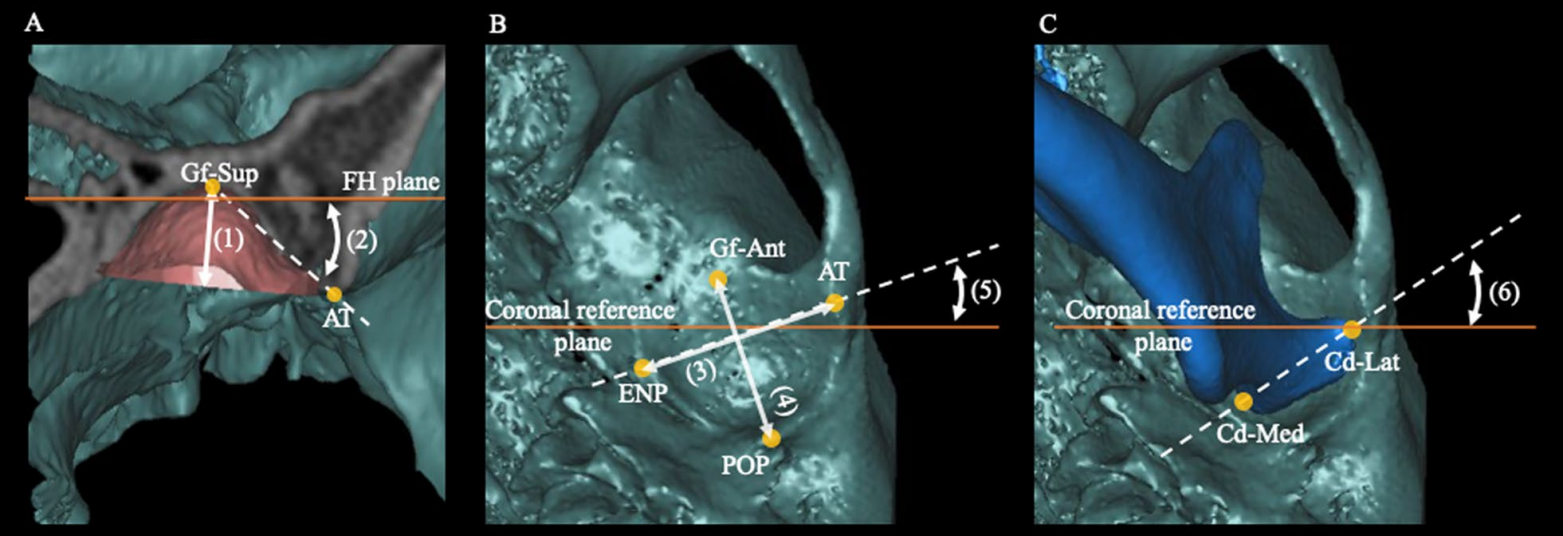
Linear and angular measurements for the evaluation of temporomandibular joint morphology. **A**: The glenoid fossa depth was defined as the perpendicular distance from the most superior point of the glenoid fossa (Gf-Sup) to the glenoid fossa base (1). The articular eminence angle (AE angle) was defined as the angle between the tangent line connecting Gf-Sup-AT and the Frankfort horizontal plane (FH) (2). **B**: The glenoid fossa width was defined as the distance between AT and ENP (3) and the glenoid fossa length was defined as the distance between POP and the most anterior point of the glenoid fossa base (4). The axial glenoid fossa angle was defined as the angle between the line connecting ENT-AT and the coronal reference plane (5). **C**: The axial condylar angle was defined as the angle between the horizontal condylar axis (Cd-Med–Cd-Lat) and the coronal reference plane (6)

**Fig. 4:**
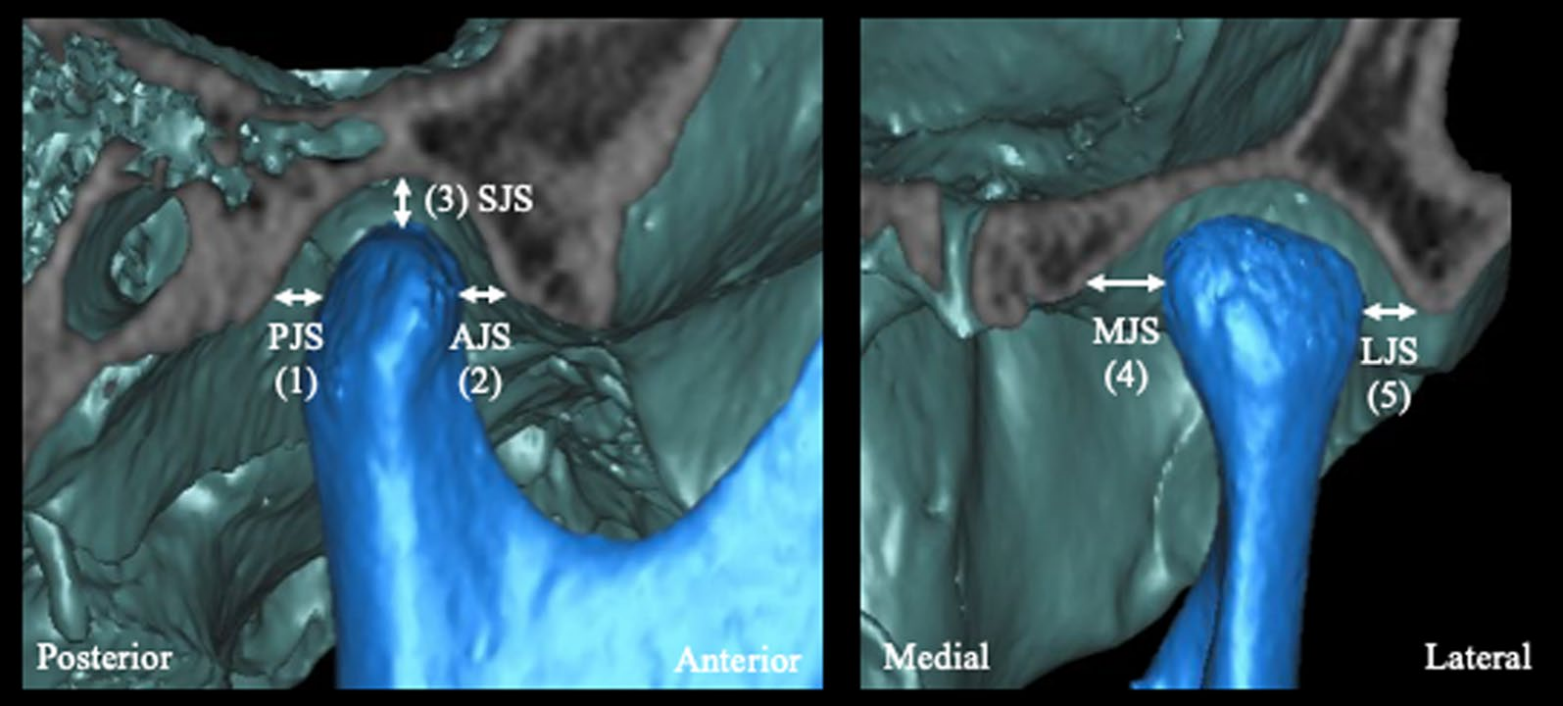
Posterior joint space (PJS) (1) and anterior joint space (AJS) (2) were defined as the distance parallel to the FH plane between the posterior and anterior points of the condyle and the glenoid fossa outline, respectively. The superior joint space (SJS) (3), medial joint space (MJS) (4) and lateral joint space (LJS) (5) were defined as the distance between the most medial point, superior point, and lateral point of the condyle and the glenoid fossa, respectively

The glenoid fossa base was defined as the plane passing through the most inferior point of the articular tubercle (AT), the most inferior point of the entoglenoid process (ENP), and the most inferior point of the postglenoid process (POP) (Table 1, Fig. 2A). The glenoid fossa volume and glenoid fossa surface area were measured for the structure above the glenoid fossa base (Table 2, Fig. 2B and 2C). The condyle volume was also defined as the volume of the condyle above the glenoid fossa base (Table 2, Fig. 2D and 2E). The glenoid fossa width, length, and depth; articular eminence angle; axial glenoid fossa angle; and axial condylar angle were evaluated to assess TMJ morphology as given in Table 2 and Fig. 3A, 2B and 2C. The TMJ spaces such as the posterior joint space (PJS), anterior joint space (AJS), superior joint space (SJS), medial joint space (MJS), and lateral joint space (LJS) were evaluated as shown in Table 2 and Fig. 4. The fossa–condyle volume ratio, in which the volume of the glenoid fossa was divided by the volume of the condyle, was also evaluated to assess the occupancy of the condyle within the glenoid fossa.

**Table 2:** Definitions of measurement of the temporomandibular joint parameters

Temporomandibular joint parameters	Definition
Glenoid fossa volume	The volume of the glenoid fossa above the fossa base plane
Glenoid fossa surface area	The surface area of glenoid fossa above the fossa base plane
Glenoid fossa width	The distance between AT and ENP
Glenoid fossa length	The distance between Gf-ant and POP
Glenoid fossa depth	The perpendicular distance of most superior point of glenoid fossa to fossa base plane
Articular eminence (AE) angle	The angle between the tangent line connecting Gf-Sup and AT, and the FH plane
Glenoid fossa axial angle	The angle between the tangent line connecting ENP and AT, and the coronal reference plane
Condylar volume	The volume of condyle above the fossa base plane
Condylar axial angle	The angle between the tangent line connecting Con-med and Con-lat, and the coronal reference plane
Fossa/condyle volume ratio	The volume of glenoid fossa divided by the volume of the condyle
Anterior joint space	The distance parallel to FH plane between the anterior point of the condyle in the glenoid fossa and the anterior wall of glenoid fossa
Posterior joint space	The distance parallel to FH plane between the posterior point of the condyle in the glenoid fossa and the posterior wall of glenoid fossa
Superior joint space	The distance between the most superior point of the condylar in the glenoid fossa and the superior wall of glenoid fossa
Lateral joint space	The distance between the most medial point of the condylar in the glenoid fossa and the medial wall of glenoid fossa
Medial joint space	The distance between the most medial point of the condylar in the glenoid fossa and the medial wall of glenoid fossa

To analyse the functional asymmetry of condylar movement, each patient was instructed to perform maximum voluntary protrusive movements with the teeth in contact, and three-dimensional condylar movement was recorded by computed axiography (CADIAX®; Gamma Dental, Klosterneuburg, Austria). The condylar path length (CPL, the distance of the most translated position of the condyle on the sagittal plane), the sagittal condylar inclination (SCI) angle on the sagittal plane, and the transverse condylar inclination (TCI) angle on the horizontal plane were measured during these movements as reported by Tun Oo et al*.* [16] and as shown in Additional file: 1 Fig. 1. For TCI, deviation towards the shifted side was defined as a negative value, while deviation towards the non-shifted side was defined as a positive value.

Side-to-side asymmetry (shifted side versus non-shifted side) in the 3D morphology of the TMJ and the condylar movement were assessed in both groups. Previous studies showed that the condyle and the glenoid fossae morphology do not significantly differ bilaterally in patients without facial asymmetry [9, 17]. Therefore, the mean value of the two sides in the control group were compared with the values of both the shifted and non-shifted sides in the MA group. Additionally, to overcome the influence of the difference in the sizes of individual faces and to focus on the extent of intersubject asymmetry, the asymmetry index [(non-shifted side − shifted side) / (non-shifted side + shifted side) × 100] was used to assess the correlation between the 3D morphology of the TMJ and condylar movement.

The CT images and CADIAX materials used for evaluating the 3D TMJ morphology and condylar movement in the current study are the same as those used by Tun Oo et al. [16]. To exclude interexaminer errors, the same investigator performed the anatomical landmark location and mandibular functional movement measurement. After a 2-week interval, the same investigator reanalysed all variables to assess the reliability of the measurements on the 3D images. The interclass correlation coefficients were calculated, with values greater than 0.8 indicating high reliability.

To compare measurements between the shifted and non-shifted sides within each group, Wilcoxon’s signed-ranked test was performed. For comparison between the two groups, the Mann–Whitney U test was used. Spearman’s rank correlation coefficient was used to analyse the correlation between the 3D morphology of the TMJ and condylar movement. SPSS version 10.0 (SPSS Inc., Chicago, IL, USA) was used for all the analyses, with the level of significance set at *P* < 0.05 and *P* < 0.01.

## Results

In this study, the intraclass correlation coefficients of the measurements ranged from 0.905 to 0.980, indicating excellent reliability. The characteristics of the patients are provided in Table 3. There was no significance difference between the two group in age, ANB° and Wits appraisal, but there was a significant difference for mention deviation (*P* < 0.001). The incidence of TMJ disorder symptoms (presence or absence of clicking sounds during condylar movements) is shown in Table 4. The percentage of the presence of clicking sounds is higher in the MA group than in the control group (80% vs 36%).

**Table 3:** Patient characteristics in the control and MA groups

	Control group		MA group		
Variable	Mean	SD	Mean	SD	*P* values
Age (years)	21.99	4.65	25.87	6.61	0.175
ANB (°)	-3.96	2.24	-2.82	2.76	0.304
MD (mm)	1.30	1.10	9.70	4.10	< 0.000**
Wits appraisal (mm)	-4.50	2.98	-4.10	3.06	0.586

*SD* Standard deviation, *MD* Degree of menton deviation

^**^P < 0.01

**Table 4:** Temporomandibular joint disorder symptoms

	Control group	MA group
Shifted side	2 (8%)	5 (20%)
Non-shifted side	5 (20%)	9 (36%)
Bilateral sides	2 (8%)	6 (24%)
Total	9 (36%)	20 (80%)

Table 5 shows a comparison of the 3D morphology of the TMJ between the shifted and non-shifted sides within each group. The TMJ morphology parameters in the control group did not differ significantly between the two sides. In the MA group, the glenoid fossa volume, glenoid fossa surface area, glenoid fossa length, and condylar volume were significantly smaller on the shifted side versus the non-shifted side (*P* < 0.001, *P* = 0.001, *P* = 0.017, and *P* < 0.001, respectively). The glenoid fossa axial angle, articular eminence angle, and condylar axial angle were significantly greater on the shifted side versus the non-shifted side (*P* = 0.001, *P* < 0.001, and *P* = 0.002 respectively). There was no significant difference between the shifted and non-shifted sides in the fossa/condyle volume ratio, or the medial, lateral, superior, anterior, and posterior joint spaces in the MA group. The results of the comparison of 3D condylar movement between the shifted and non-shifted sides in the control group and MA group are provided in Additional file: 2  Table 1.

**Table 5:** Comparison of temporomandibular joint morphology on the shifted and non-shifted sides within the control group and the MA group

Measurement	Control group					MA group				
	Shifted side		Non-shifted side		*P*-value	Shifted side		Non-shifted side		*P*-value
	Mean	SD	Mean	SD		Mean	SD	Mean	SD	
Glenoid fossa volume	2194.65	399.92	2171.18	3338.44	0.459	1884.67	340.93	2239.35	371.27	< 0.000**
Glenoid fossa surface area	1159.83	130.33	1142.00	137.69	0.313	1063.02	100.77	1178.98	118.72	0.001**
Glenoid fossa width	27.72	3.07	27.49	2.79	0.819	26.83	2.07	27.16	1.96	0.628
Glenoid fossa length	19.08	1.82	18.79	1.63	0.353	17.95	1.37	18.88	1.66	0.017*
Glenoid fossa depth	9.39	1.46	9.08	1.25	0.236	9.56	1.48	9.12	1.42	0.129
Glenoid fossa Angle	19.16	4.52	19.12	4.09	0.957	23.05	5.13	19.43	5.32	0.001**
Articular eminence Angle	31.49	5.25	31.47	5.07	0.581	34.49	3.59	30.82	4.75	< 0.000**
Condylar volume	708.08	188.14	717.84	173.54	0.619	565.68	142.18	702.56	121.12	< 0.000**
Condylar angle	21.64	2.44	21.33	3.71	0.653	24.01	3.50	20.31	9.83	0.002**
Fossa/ condyle volume ratio	3.38	0.71	3.35	0.62	0.427	3.14	1.00	3.26	0.76	0.332
Medial joint space	2.68	0.28	2.88	0.32	0.638	2.23	0.38	2.38	0.41	0.283
Lateral joint space	2.73	0.38	2.93	0.41	0.186	2.54	0.29	2.71	0.43	0.297
Superior joint space	2.17	0.67	2.23	0.70	0.419	1.65	0.27	1.80	0.31	0.281
Anterior joint space	2.66	0.85	2.62	0.79	0.449	2.35	0.32	2.21	0.60	0.251
Posterior joint space	2.24	0.66	2.24	0.74	0.211	2.25	0.38	2.17	0.41	0.484

*SD* Standard deviation

^*^P < 0.05, **P < 0.01

Table 6 shows the mean values of the 3D TMJ morphology on both sides in the control group and on the shifted and non-shifted sides in the MA group. Compared with the average values of the 3D TMJ morphology in the control group, the glenoid fossa volume, glenoid fossa surface area, glenoid fossa length, and condyle volume on the shifted side in the MA group were significantly smaller (*P* = 0.011, *P* = 0.028, *P* = 0.025, and *P* = 0.002, respectively). The glenoid fossa axial angle, articular eminence angle, and condylar axial angle on the shifted side in the MA group were significantly greater when compared with the average values of the control group (*P* = 0.013, *P* = 0.018, and *P* = 0.003 respectively). The 3D morphology of the glenoid fossa and condyle on the non-shifted side in the MA group did not differ significantly from that of the control group. Compared with the mean values of the control group, the medial, superior, and anterior joint spaces in the MA group were significantly smaller on both the shifted and non-shifted sides (MJS; *P* = 0.005 and *P* = 0.013, SJS; *P* = 0.007 and *P* = 0.019 and AJS; *P* = 0.012 and *P* = 0.025).

**Table 6:** Comparison of temporomandibular joint morphology on the shifted and non-shifted sides of the MA group and mean values of both sides of the control group

Measurement	Control group		MA group					
	Mean values of both sides		Shifted side		*P*-value	Non-shifted side		*P*-value
	Mean	SD	Mean	SD		Mean	SD	
Glenoid fossa volume	2182.92	352.68	1884.67	340.93	0.011*	2239.35	371.27	0.516
Glenoid fossa surface area	1150.91	126.27	1063.02	100.77	0.028*	1178.98	118.72	0.171
Glenoid fossa width	27.60	2.49	26.83	2.07	0.117	27.16	1.96	0.503
Glenoid fossa length	18.93	1.50	17.95	1.37	0.025*	18.88	1.66	0.463
Glenoid fossa depth	9.23	1.22	9.56	1.48	0.352	9.12	1.42	0.669
Glenoid fossa Angle	19.14	4.24	23.05	5.13	0.013*	19.43	5.32	0.528
Articular eminence Angle	31.48	4.35	34.49	3.59	0.018*	30.82	4.75	0.691
Condylar volume	712.96	166.72	565.68	142.18	0.002**	702.56	121.12	0.778
Condylar angle	21.48	2.57	24.01	3.50	0.003**	20.31	9.83	0.367
Fossa/ condyle volume ratio	3.36	0.61	3.14	1.00	0.177	3.26	0.76	0.778
Medial joint space	2.78	0.67	2.23	0.38	0.005**	2.38	0.41	0.013*
Lateral joint space	2.83	0.35	2.54	0.29	0.365	2.71	0.43	0.268
Superior joint space	2.20	0.65	1.65	0.27	0.007**	1.80	0.31	0.019*
Anterior joint space	2.68	0.85	2.35	0.32	0.016*	2.21	0.60	0.030*
Posterior joint space	2.33	0.22	2.25	0.38	0.259	2.17	0.41	0.352

*SD* Standard deviation

^*^P < 0.05, **P < 0.01

**Fig. 5:**
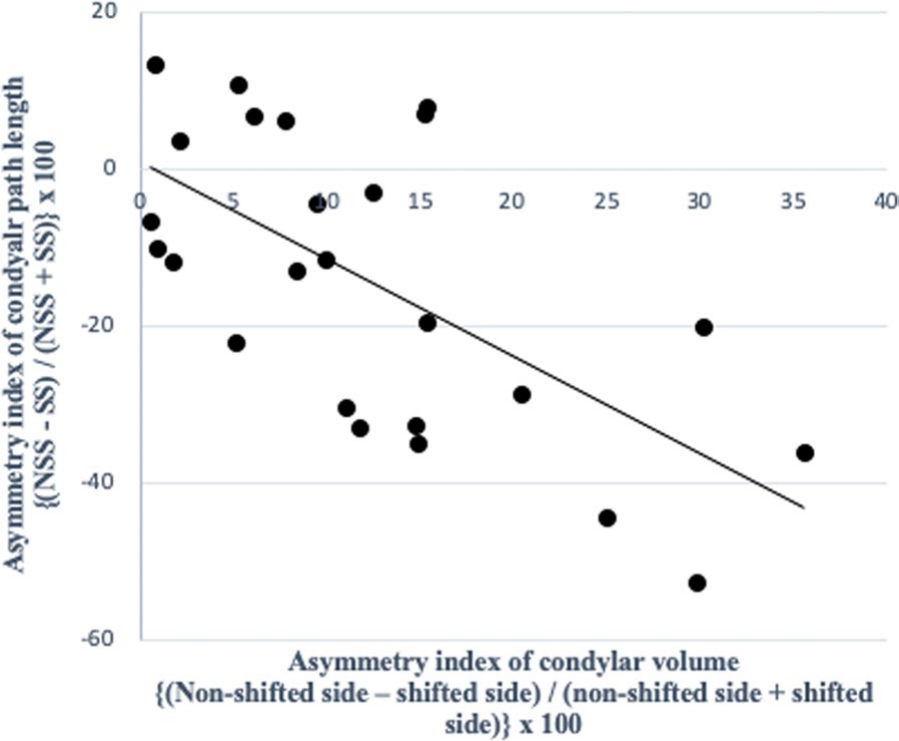
Simple regression analysis of the asymmetry index of the condylar volume and the condylar path length in the mandibular asymmetry group. *X*-axis, asymmetry index of the condylar volume; *Y*-axis, asymmetry index of the condylar path length

**Fig. 6:**
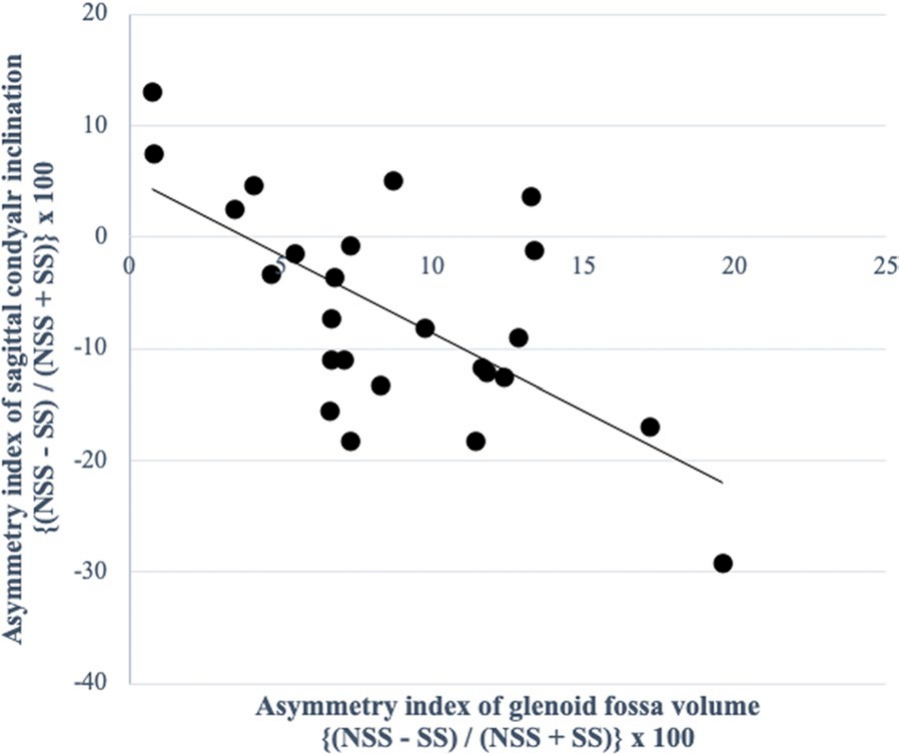
Simple regression analysis of the asymmetry index of the glenoid fossa volume and the sagittal condylar inclination in the mandibular asymmetry group. *X*-axis, asymmetry index of the glenoid fossa volume; *Y*-axis, asymmetry index of the sagittal condylar inclination

**Fig. 7:**
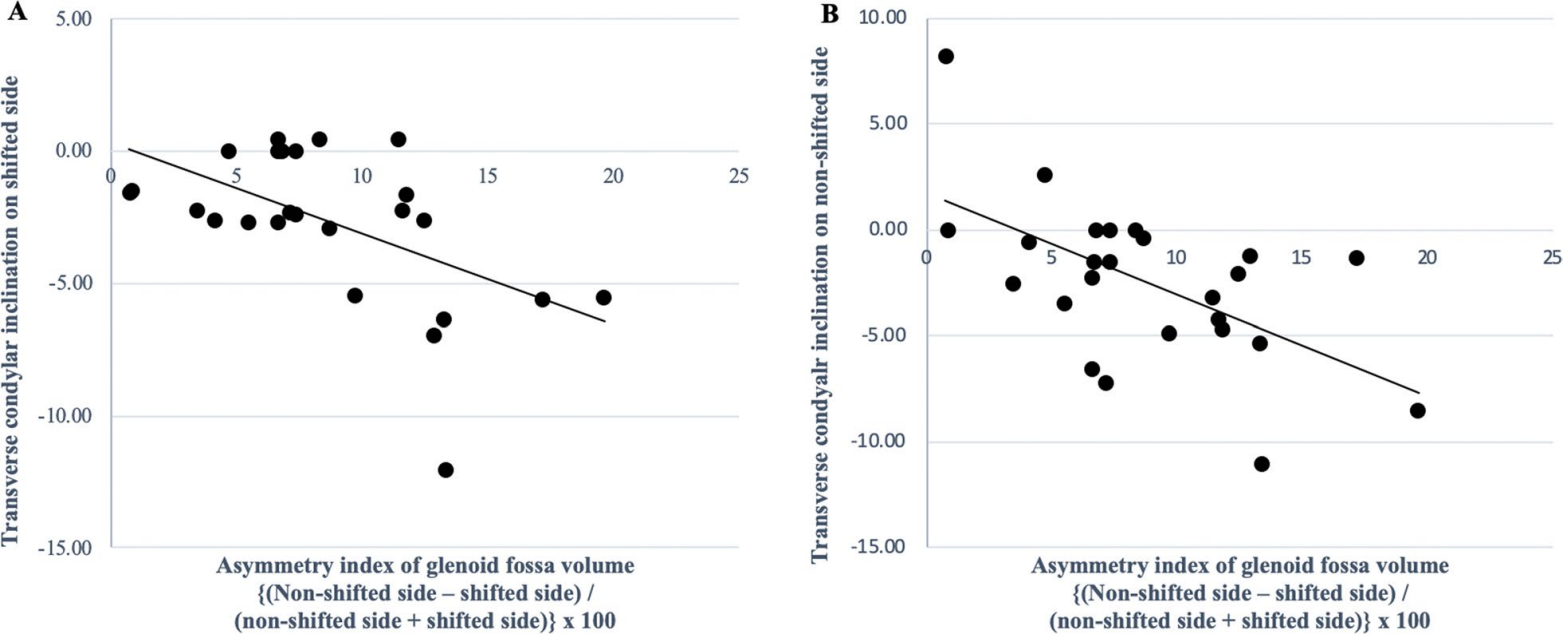
Simple regression analysis of the asymmetry index of the glenoid fossa volume and the transverse condylar inclination in the mandibular asymmetry group. **A**: Relationship with the transverse condylar inclination on the shifted side. *X*-axis, asymmetry index of the glenoid fossa volume; *Y*-axis, transverse condylar inclination on the shifted side. **B**: Relationship with the transverse condylar inclination on the non-shifted side. *X*-axis, asymmetry index of the glenoid fossa volume; *Y*-axis, transverse condylar inclination on the non-shifted side

**Fig. 8:**
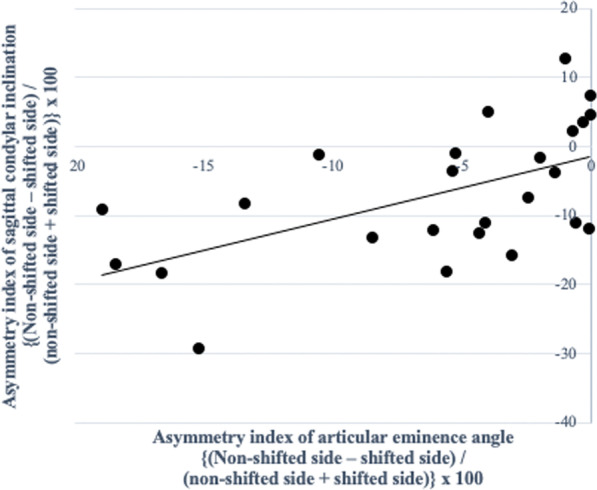
Simple regression analysis of the asymmetry index of the articular eminence angle and the sagittal condylar inclination in the mandibular asymmetry group. *X*-axis, asymmetry index of the articular eminence angle; *Y*-axis, asymmetry index of the sagittal condylar inclination

In the MA group, there were significant negative correlations between the asymmetry index of the condylar volume with CPL (*P* = 0.005, *r* = -0.548) (Fig. 5; Table 7), and the glenoid fossa volume with the SCI (*P* = 0.009, *r* = -0.510) (Fig. 6; Table 7). The asymmetry index of the glenoid fossa volume was also negatively correlated with the TCI on the shifted side (*P* = 0.006, *r* =  − 0.536) and the non-shifted side (*P* = 0.016, *r* =  − 0.478), indicating that as the left–right asymmetry of the glenoid fossa volume increased, both condyles in the MA group tended to slide towards the shifted side (Figs. 7A and 7B; Table 7). The asymmetry index of the articular eminence angle was positively correlated with the SCI asymmetry index (*P* = 0.002, *r* = 0.593), indicating that the left–right asymmetry of the articular eminence increased in tandem with the left–right asymmetry of the SCI (Fig. 8; Table 7).

**Table 7:** Correlation between the asymmetry index values of temporomandibular joint morphology and condylar movement in the MA group

Measurement	Mean	MA group	Correlation with the CPL asymmetry index (P-value)	Correlation with the SCI asymmetry index (P-value)	Correlation with the TCI SS (*P*-value)	Correlation with the TCI nSS (*P*-value)
		SD				
Glenoid fossa volume	1.20	0.11	0.119	0.009**	0.006**	0.017*
Glenoid fossa surface area	1.11	0.10	0.679	0.489	0.163	0.168
Glenoid fossa width	1.02	0.09	0.531	0.145	0.178	0.714
Glenoid fossa length	1.06	0.09	0.416	0.342	0.388	0.751
Glenoid fossa depth	0.96	0.12	0.390	0.798	0.672	0.711
Glenoid fossa Angle	0.85	0.23	0.326	0.836	0.542	0.652
Articular eminence Angle	0.89	0.10	0.633	0.002**	0.307	0.616
Condylar volume	1.29	0.32	0.005**	0.839	0.058	0.080
Condylar angle	0.80	0.46	0.458	0.512	0.581	0.694
Fossa/ condyle volume ratio	0.97	0.23	0.399	0.145	0.981	0.501
Medial joint space	1.05	0.14	0.276	0.696	0.203	0.088
Lateral joint space	1.08	0.21	0.180	0.475	0.207	0.421
Superior joint space	1.11	0.23	0.198	0.698	0.231	0.167
Anterior joint space	1.21	0.44	0.445	0.276	0.261	0.102
Posterior joint space	1.07	0.28	0.981	0.123	0.166	0.499

Asymmetry index = (shifted side – non-shifted side) / (shifted side + non-shifted side) × 100. *SD* Standard deviation;

*CPL* Condylar path length, *SCI* Sagittal condylar inclination, *TCI SS* Transverse condylar inclination on shifted side, *TCI nSS* Transverse condylar inclination on non-shifted side

^*^P < 0.05, **P < 0.01

## Discussion

Studies have evaluated the morphological asymmetry of craniofacial structures to provide better treatment for MA patients. However, functional asymmetry related to masticatory dysfunction is also reported, and there is still limited information concerning the relationship between the asymmetry of skeletal structures and function in these patients. It is important to unravel the mechanism of MA to comprehensively understand the developmental process of masticatory dysfunction and to establish evidence-based occlusal treatment for MA patients.

In the MA group, the volume of both the condyle and the glenoid fossa on the shifted side was significantly smaller than that of the non-shifted side. This result is similar to previous morphological evaluations [9, 18]. The current study also found that the anteroposterior length and surface area of the glenoid fossa on the shifted side were significantly smaller than those of the non-shifted side, with no difference in the mediolateral width of the glenoid fossa. A previous study involving 3D comparison of the shape and size of the TMJ of facial asymmetry patients also reported that the glenoid fossa on the shifted side was significantly smaller on the sagittal view compared with that of the non-shifted side, but with no difference in the coronal view [19]. In the MA group, although the morphology of the condyles and the glenoid fossa differed bilaterally, there was no significant difference in the condyle–fossa ratio, indicating that the occupancy of the condyle within the glenoid fossa is the same bilaterally. Additionally, the measurements of the TMJ spaces were similar bilaterally, consistent with the findings of a previous study [20]. Therefore, in MA patients, as the mandible grows asymmetrically with a smaller condyle on the shifted side, the TMJ structure of the glenoid fossa may also undergo remodelling to maintain the same condyle–fossa relationship. The articular eminence angle was also significantly different between the shifted and non-shifted sides, with the shifted side angle being steeper. This difference in the articular eminence angle may be derived from an adaptation to asymmetrical loading to the TMJ [21]. The discussion on the comparison of 3D condylar movement between the shifted and non-shifted sides in the MA and the control groups can be found in the supplementary. (Additional file: 3).

Previous studies have reported that untreated malocclusion with a unilateral posterior crossbite causes yawing of the mandible towards the crossbite side and posterior displacement of the condyle and glenoid fossa on the crossbite side [22]. This may lead to deviation and asymmetrical growth of the mandible [22]. Our previous study also observed posterior displacement of the glenoid fossa on the shifted side; this could be an etiological factor for asymmetrical development of the mandible in MA patients [16]. With yawing of the mandible caused by posterior displacement of the condyle and the glenoid fossa on the shifted side, outward rotation of the condylar angle may be increased on the shifted side, worsening the symptoms of the mandibular shift. The results of the current study showed that the axial condylar angle and the glenoid fossa angle were significantly larger on the shifted sides. Therefore, it would be reasonable to assume that as the mandible grows asymmetrically with the condyle and glenoid fossa displaced posteriorly towards the shifted side, the condyle on the shifted side would adjust and rotate inwardly along with the glenoid fossa to prevent worsening of the symptoms of the mandibular shift and to compensate for the asymmetrical development of the mandible in patients with MA.

Compared with the control group, the TMJ morphology of both the condyle and the glenoid fossa on the shifted side was significantly different in the MA group. This is consistent with the results of Ikeda et al. [9] but not with those of Kim et al. [18]. The differences may relate to patient selection; for example, the inclusion or exclusion of skeletal Class II patients, in whom the morphology of the condyles and glenoid fossa differs to that of others skeletal Class patients [14]. In the comparison of TMJ spaces between the two groups, the medial, anterior, and superior joint spaces of both sides of the MA group were significantly smaller than those of the control group, as found in previous studies [17, 20]. It has been suggested that the reduction in joint space may lead to severe squeezing of the articular disc within the TMJ, which in turn may increase the biomechanical load on the TMJ and increase the risk of TMJ disorder [17, 20]. Therefore, this may be related to a higher percentage of patients with clicking sounds in the MA group than in the control group when evaluating the clinical signs of TMJ disorder.

A previous study reported that mandibular structural asymmetry (of the condyle, ramus, and mandibular body) was closely related to asymmetrical condylar movement [9]. In the current study, the condylar volume above the glenoid fossa base was evaluated, and this condylar volume asymmetry was negatively correlated with the asymmetry of the CPL in the MA group. The smaller the condylar volume was on the shifted side, the longer was the CPL on the shifted side. The asymmetry of condylar movement may compensate for the asymmetry of the condyle morphology.

The asymmetry of the glenoid fossa volume was negatively correlated with the asymmetry of the SCI angle. The smaller the glenoid fossa on the shifted side, the greater was the SCI angle on the shifted side. Additionally, the asymmetry of the glenoid fossa volume was also found to be negatively correlated with TCI angles on both sides with negative values. Therefore, as the morphological asymmetry of the glenoid fossa increased, the condyles on both sides tended to move towards the shifted side on protrusive movement in MA patients. A previous report comparing magnetic resonance imaging and computed axiography in patients with TMJ disorder also speculated that the 3D condylar movement asymmetry could be caused by functional compensation in the anatomical asymmetry of the TMJ [23]. Moreover, another previous study in patients with unilateral crossbite speculated that there was a relationship between morphological and functional asymmetry because they noted that the articular eminence angle was steeper on the crossbite side than the other side, as was the condylar path angle [24]. Additionally, the current study also showed that the asymmetrical steepness of the articular eminence angle was positively correlated with the asymmetry of the SCI angle, indicating that the steeper the articular eminence angle was on the shifted side, the greater was the SCI angle on the shifted side. Because sliding condylar movements are observed along the anatomical form of the articular eminence [25], the same can be said for MA patients in whom the asymmetrical sagittal condylar angle of condylar movement on protrusion closely follows the morphological asymmetry of the articular eminence angle. Therefore, 3D morphological asymmetry of the glenoid fossa is closely related to the functional asymmetry of condylar movement in MA patients.

The current study in MA patients showed that asymmetrical condylar morphology significantly correlates with an asymmetrical condylar path length, and that asymmetrical glenoid fossa morphology significantly correlates with an asymmetrical sagittal steepness angle of condylar movement. Therefore, the asymmetrical changes in the 3D morphology of the TMJ (both the condyle and glenoid fossa) closely interact with masticatory dysfunction in asymmetrical condylar movements and affect the development of mandibular asymmetry. From a clinical viewpoint, a close relationship between asymmetry of the TMJ structure and condylar movement in MA patients could indicate the need for early treatment of asymmetrical condylar movements that result in a functional shift of the mandible at an early age. Moreover, in MA patients, given that functional asymmetry of condylar movement is reported to remain after surgery [8] and that there is a close relationship between the asymmetry of the TMJ morphology and function, these asymmetries and their reciprocal relationships might affect the stability of the treatment. Further research is needed to follow up MA patients after surgery.

There were several limitations in the current study. The articular disc position, which may affect condylar movement, was not evaluated. Additionally, the TMJ undergoes a remodelling process throughout life, and there may be long-term changes in the masticatory function of condylar movement that is affected by the TMJ morphology. Therefore, longitudinal observation of both condylar movement and TMJ morphology may be necessary.

## Conclusion

In patients with MA, the morphology of the condyles and glenoid fossae were asymmetrical, and this asymmetry was closely related to functional asymmetry of condylar movement. Proving that morphological asymmetry of TMJ is closely related to condylar movement in MA patients could indicate the need for early treatment of asymmetrical condylar movements that result in a functional shift of the mandible at an early age.

## Supplementary Information

**Additional file 1: Fig. S1. **Schematic representation of the condylar path in mandibular protrusive movements and the measurement parameters for condylar path analysis. The condylar path length (CPL) was measured as the shortest linear distance between the reference point (RP) and the most translated position of the condyle in the sagittal plane. Sagittal condylar inclination (SCI) and transverse condylar inclination (TCI) were measured 5 mm from the RP in the sagittal and horizontal planes, respectively. Reproduced with permission from the European Journal of Orthodontics, Tun Oo et al. [16].

**Additional file 2: Table S1. **Comparison of condylar movement on the shifted and non-shifted sides in the control and mandibular asymmetry groups.The MA group showed that CPL was significantly longer, and SCI was significantly steeper, on the shifted side versus the non-shifted side (P = 0.003 and P = 0.001), while TCI did not show a significant difference. The TCI on both the shifted and non-shifted sides in the MA group showed negative values. Reproduced with permission from the European Journal of Orthodontics, Tun Oo et al. [16].

**Additional file 3: **In the current study, 3D condylar movement was evaluated by computed axiography and showed that CPL was significantly longer, and SCI was significantly steeper, on the shifted side versus the non-shifted side in the MA group. Previous studies that evaluated jaw movement in MA patients using an optical tracking camera system [8] and computed axiography [7, 9] also showed that condylar movement on the shifted side was greater than that on the contralateral side. In the MA group, the TCI on both the shifted and non-shifted sides showed negative values, indicating that bilateral condyles tended to move towards the shifted side during protrusive movements in MA patients. Ishizaki et al. [7] reported that the condyle on the shifted side tended to move outward during open–close and protrusion–retrusion movements. Fushima et al. [26] suggested that condylar movement in the horizontal plane on one side affects the movement of the contralateral side during symmetrical condylar movement. These findings are consistent with the values for the TCI in the current study.

## Data Availability

The data underlying this article are available.

## Abbreviations

3D: Three-dimensional
AE angle: The articular eminence angle
AJS: Anterior joint space
AT: Articular tubercle
CPL: Condylar path length
CT: Computed tomography
FH: Frankfort horizontal plane
Gf-Sup: The most superior point of the glenoid fossa
LJS: Lateral joint space
MA: Mandibular asymmetry
MJS: Medial joint space
PJS: Posterior joint space
POP: The most inferior point of the postglenoid process
RP: Reference point
SCI: Sagittal condylar inclination
SJS: Superior joint space
TCI: Transverse condylar inclination
TMJ: Temporomandibular joint
