# Supplementary figures and images for: Three-dimensional characteristics of temporomandibular joint morphology and condylar movement in patients with mandibular asymmetry
Source: Prog Orthod. 2022 Dec 29;23:50. doi: 10.1186/s40510-022-00445-0 (PMC9797632; doi:10.1186/s40510-022-00445-0)

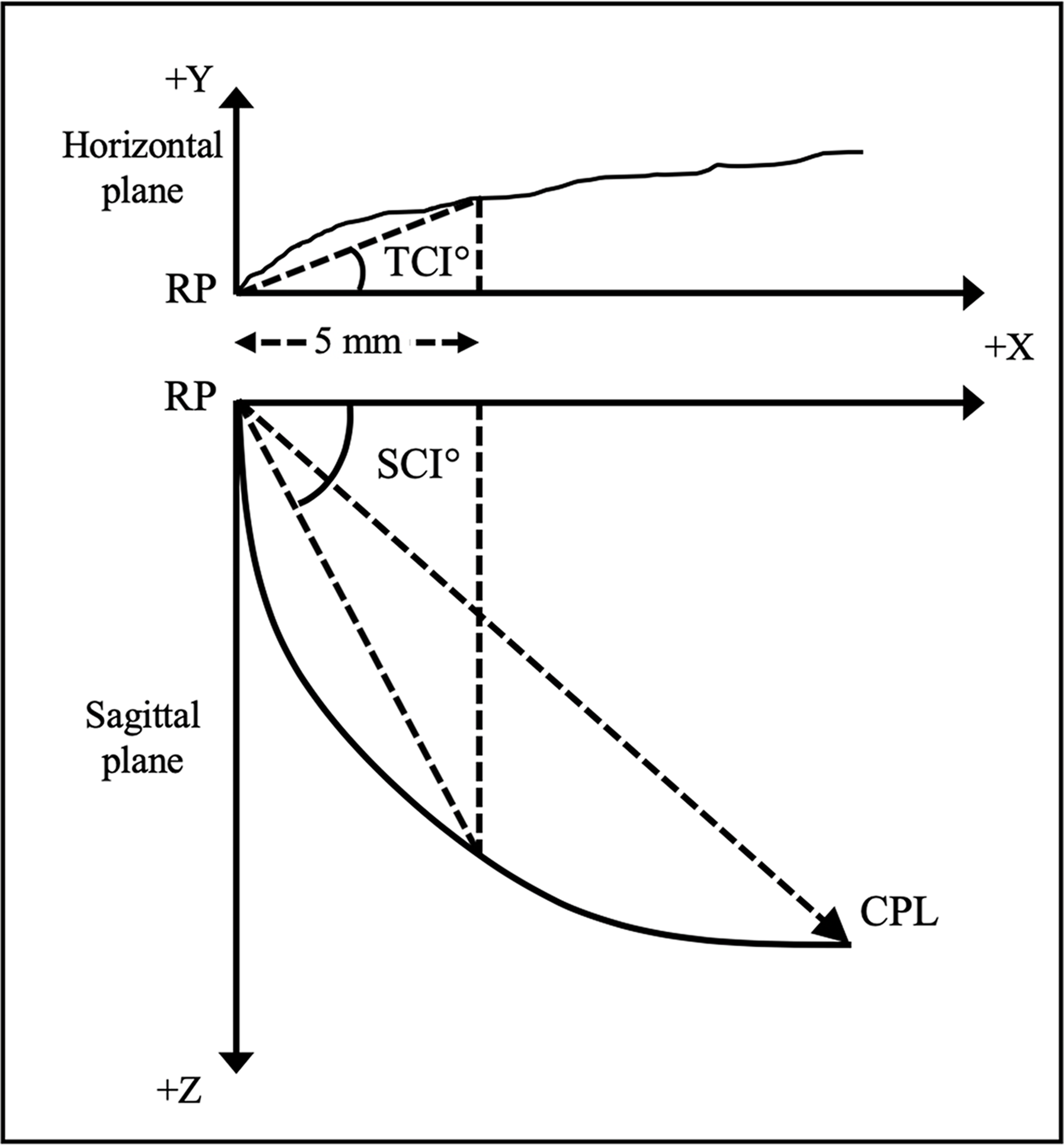

Supplement: Supplementary file 1 — Additional file 1: Fig. S1. Schematic representation of the condylar path in mandibular protrusive movements and the measurement parameters for condylar path analysis. The condylar path length (CPL) was measured as the shortest linear distance between the reference point (RP) and the most translated position of the condyle in the sagittal plane. Sagittal condylar inclination (SCI) and transverse condylar inclination (TCI) were measured 5 mm from the RP in the sagittal and horizontal planes, respectively. Reproduced with permission from the European Journal of Orthodontics, Tun Oo et al. [16]. [file 40510_2022_445_MOESM1_ESM.tif]
