# Supplementary material for: Three-dimensional characteristics of temporomandibular joint morphology and condylar movement in patients with mandibular asymmetry
Source: Prog Orthod. 2022 Dec 29;23:50. doi: 10.1186/s40510-022-00445-0 (PMC9797632; doi:10.1186/s40510-022-00445-0)
Supplement: Supplementary file 2 — Additional file 2: Table S1. Comparison of condylar movement on the shifted and non-shifted sides in the control and mandibular asymmetry groups.The MA group showed that CPL was significantly longer, and SCI was significantly steeper, on the shifted side versus the non-shifted side (P = 0.003 and P = 0.001), while TCI did not show a significant difference. The TCI on both the shifted and non-shifted sides in the MA group showed negative values. Reproduced with permission from the European Journal of Orthodontics, Tun Oo et al. [16]. [file 40510_2022_445_MOESM2_ESM.docx]

Table 7. Comparison of the condylar movement on the shifted and non-shifted sides in the control and MA groups.

|  | Control group | | | |  | MA group | | | |  |
| --- | --- | --- | --- | --- | --- | --- | --- | --- | --- | --- |
|  | Shifted side | | Non-shifted side | |  | Shifted side | | Non-shifted side | |  |
| Measurement | Mean | SD | Mean | SD | P-value | Mean | SD | Mean | SD | P-value |
| CPL (mm) | 5.45 | 2.16 | 5.60 | 2.17 | 0.893 | 7.52 | 3.14 | 5.52 | 2.24 | 0.003^**^ |
| SCI (°) | 41.08 | 8.08 | 41.91 | 5.98 | 0.339 | 44.72 | 8.18 | 39.03 | 7.12 | 0.001^**^ |
| TCI (°) | -0.48 | 3.91 | 0.42 | 6.55 | 0.648 | -2.70 | 2.29 | -2.43 | 3.82 | 0.840 |

CPL, condylar path length; SCI, sagittal condylar inclination; TCI, transverse condylar inclination; SD, standard deviation.

**P < 0.01
