# Supplementary material for: Three-dimensional characteristics of temporomandibular joint morphology and condylar movement in patients with mandibular asymmetry
Source: Prog Orthod. 2022 Dec 29;23:50. doi: 10.1186/s40510-022-00445-0 (PMC9797632; doi:10.1186/s40510-022-00445-0)
Supplement: Supplementary file 3 — Additional file 3: In the current study, 3D condylar movement was evaluated by computed axiography and showed that CPL was significantly longer, and SCI was significantly steeper, on the shifted side versus the non-shifted side in the MA group. Previous studies that evaluated jaw movement in MA patients using an optical tracking camera system [8] and computed axiography [7, 9] also showed that condylar movement on the shifted side was greater than that on the contralateral side. In the MA group, the TCI on both the shifted and non-shifted sides showed negative values, indicating that bilateral condyles tended to move towards the shifted side during protrusive movements in MA patients. Ishizaki et al. [7] reported that the condyle on the shifted side tended to move outward during open–close and protrusion–retrusion movements. Fushima et al. [26] suggested that condylar movement in the horizontal plane on one side affects the movement of the contralateral side during symmetrical condylar movement. These findings are consistent with the values for the TCI in the current study. [file 40510_2022_445_MOESM3_ESM.docx]

Supplementary discussion

In the current study, 3D condylar movement was evaluated by computed axiography and showed that CPL was significantly longer, and SCI was significantly steeper, on the shifted side versus the non-shifted side in the MA group. Previous studies that evaluated jaw movement in MA patients using an optical tracking camera system (7) and computed axiography (6, 8) also showed that condylar movement on the shifted side was greater than that on the contralateral side. In the MA group, the TCI on both the shifted and non-shifted sides showed negative values, indicating that bilateral condyles tended to move towards the shifted side during protrusive movements in MA patients. Ishizaki et al*.* (6) reported that the condyle on the shifted side tended to move outward during open–close and protrusion–retrusion movements. Fushima et al*.* (23) suggested that condylar movement in the horizontal plane on one side affects the movement of the contralateral side during symmetrical condylar movement. These findings are consistent with the values for the TCI in the current study.

**References**

(6) Ishizaki K, Suzuki K, Mito T, Tanaka EM and Sato S. (2010) Morphologic, functional, and occlusal characterization of mandibular lateral displacement malocclusion. Am J Orthod Dentofacial Orthop 137, 454.e1-9. <https://doi.org/10.1016/j.ajodo.2009.10.031>

(7) Hashimoto T, Kuroda S, Lihua E, Tanimoto Y, Miyawaki S and Takano-Yamamoto T. (2008) Correlation between craniofacial and condylar path asymmetry. Journal of Oral and Maxillofacial Surgery, 66, 2020–2027. <https://doi.org/10.1016/j.joms.2008.06.003>

(8) Ikeda M, Miyamoto JJ, Takada J, Moriyama K. Association between 3-dimensional mandibular morphology and condylar movement in subjects with mandibular asymmetry. Am J Orthod Dentofac Orthop 2017;151(2):324-334. <https://doi.org/10.1016/j.ajodo.2016.06.042>

(23) Fushima K, Sato S, Suzuki Y, Kashima I, Farha K. Horizontal condylar path in patients with disk displacement with reduction. Cranio® 1997;12:78-87. <https://doi.org/10.1080/08869634.1994.11677999>
